# Match running performance is similar in lower and higher competitive standards of Spanish professional soccer accounting for effective playing time

**DOI:** 10.5114/biolsport.2024.132993

**Published:** 2023-12-20

**Authors:** Carlos Lago-Peñas, Tomás García-Calvo, Roberto López del Campo, Ricardo Resta, José Carlos Ponce-Bordón

**Affiliations:** 1 Faculty of Education and Sport Sciences, University of Vigo, Pontevedra, Spain; 2 Faculty of Sport Sciences, University of Extremadura, Cáceres, Spain; 3 Department of Competitions and Mediacoach, LaLiga, Madrid, Spain

**Keywords:** Football, Fatigue, Effective playing time, Physical demands

## Abstract

This study aimed to compare match running performance of players in the top two competitive standards of Spanish professional soccer, accounting for effective playing time (the duration of play after subtracting the game interruptions). A total of 2,784 match observations from 44 teams competing in the Spanish First Division (LaLiga Santander) and the Second Division (LaLiga Smartbank) were undertaken during two consecutive seasons (from 2021/22 to 2022/23). Total distance (TD), medium-speed running (MSR, distance 14.1–21 km · h^−1^), high-speed running (HSR, > 21 km · h^−1^), very high-speed running (VHSR, 21.1–24 km · h^−1^) and sprinting speed running distance (Sprint, > 24 km · h^−1^) were analyzed using a computerized tracking system (TRACAB, Chyronhego, New York, NY). These physical performance variables were calculated for both total and effective playing time. The main results showed that the mean effective playing time was significantly higher in matches of the First Division than in the Second Division (*p* < .01). In contrast to those observed when total playing time was considered, there were no significant differences (*p* > .05) between both competitive standards on medium speed running (MSR), high speed running (HSR), very high-speed running (VHSR), and sprint distances when the effective playing time was considered. Such findings demonstrate that contrary to previous research match running performance of players was similar in lower and higher competitive standards. Thus, effective playing time should be taken into account when interpreting the match running performance of professional soccer players.

## INTRODUCTION

The match running performance of professional and amateurs’ soccer players has been extensively studied over the past three decades. One of the most robust findings is that players at a higher standard of play perform more high-intensity running than peers at lower standards. For instance, Mohr et al. [1] found 28% and 58% more high-intensity running and sprinting in Italian elite players compared to sub-elite Danish players. Similarly, a greater distance covered in high-speed running has been observed in top versus middle and bottom ranking Danish teams [2].

However, to the best of our knowledge, very few studies have attempted to compare match running performances across different competitive standards of elite soccer within a single country and their results are contradictory. Additionally, it should be highly noted that the effective playing time was not considered in previous studies. While Bradley et al. [3] and Di Salvo et al. [4] found that players in lower standards of English soccer cover more total distance and high-intensity running during matches compared to those in the highest standard, other studies suggested the opposite [5–8].

These contradictory findings may be due to two different factors. Firstly, match running performance may be affected by the style of coaching and management, the characteristics of the players, team formation, and philosophy of play based on tradition of each country. That is why it is very interesting to study the physical performance within each country. More importantly, the argument of the current study is that the differences in players’ match running performance in different competitive standards is nonexistent or at least substantially influenced by differences in the effective playing time of games. Effective playing time is defined as the duration of play after subtracting the time taken up by stoppages, substitutions, goals, injuries, and other incidences [9, 10]. A study of game interruptions on elite soccer showed that there is an average of 108 interruptions per match and that matches are halted on average for 38% of the total match time [11]. Stoppages during a game have a significant impact on a player’s performance. Previous studies have found that stoppages cause extended extra time and reduce players’ running performance [12]; moreover, running distance in the second half of a game may decrease because of too many stoppages rather than a decrease in physical capacity [13].

Interestingly, a recent UEFA report found that the effective playing time in matches of the highest standards is higher than in lower levels and this may affect match running performances [14]. In addition, various studies have reported the amount of the effective playing time within competitive leagues [12, 13, 15, 16]. According to these studies, there exist differences in the amount of effective playing time between different leagues.

However, despite this relevant consideration, none of the aforementioned studies have compared the running performance of players across different competitive standards considering the effective playing time of games. To achieve a comprehensive analysis of the real physical demands of official soccer matches, practitioners should take into account the effective playing time, as variations on the workload of the players could not only be linked to fatigue or the level of the players. For example, Altman et al. [17] found that players covered on average 10% more total distance and performed 13% more accelerations, while sprinting 7–10% less in matches with long (> 65 min.) compared to short (< 50 min.) effective playing time. Additionally, Jerome et al. [18] suggest that the majority of sprinting distance (97%) is covered during ball-in-play.

Therefore, the aim of the current study was to compare match running performance of players in the top two competitive standards of Spanish professional soccer considering the effective playing time of matches. The research hypothesis is that differences in players’ match running performance in different competitive standards is non-existent or at least substantially influenced by differences in the effective playing time of games.

## MATERIALS AND METHODS

### Sample

The sample comprised 2,784 match observations from 44 teams competing in the First (LaLiga Santander; *n* = 1,216 records) and Second (LaLiga Smartbank; *n* = 1,568 records) Spanish soccer leagues over two consecutive seasons (from 2021/22 to 2022/23). Teams were classified according to their final league rankings. The classification of final league rankings was determined using 5 Tiers [19]: (A) 1^st^–4^th^ ranking (*n* = 244 match observations), (B) 5^th^–7^th^ ranking (*n* = 183 match observations), (C) 8^th^–17^th^ ranking (*n* = 497 match observations), (D) 18^th^–20^th^ ranking (*n* = 309 match observations), and (E) Second Division teams (*n* = 1,551 match observations). Data were retrieved from the Spanish Professional Football League (LaLiga), which allowed the use of the variables included in this investigation. In accordance with the ethical guidelines of LaLiga, this investigation does not include information that identifies soccer players (General Assembley of LaLiga, 2019). The study received the Bioethics Committee’s approval from the first author´s university (application number 239/2019).

### Procedure

Match running performance was recorded using a multicamera computerized optical tracking system TRACAB (ChryronHego VID, New York, NY), managed from the application Mediacoach (LaLiga, Madrid, Spain) that has a sampling frequency of 25 Hz. The validity and reliability of this system for the variables used have previously been investigated [20–22] and reported strong correlations (*r* > .80) and high intraclass correlation coefficients (*r* > .75) between Mediacoach multicamera tracking system and Global Positioning System. In addition, small (< .30) to moderate (> .60) SEs of estimate were observed in all speed categories used in this study.

### Data Preparation and Variables

Match running performance was divided into the following categories: total distance covered by teams in meters (TD); medium-speed running (MSR, distance 14.1–21 km · h^−1^), high-speed running (HSR, > 21 km · h^−1^), very high-speed running (VHSR, 21.1–24 km · h^−1^), and sprinting speed running distance (Sprint, > 24 km · h^−1^). All physical performance variables were calculated as i) absolute distances during total playing time in meters (m), and ii) rates of distance covered during effective playing time and normalized to meters per unit of time (m · min^−1^; [12, 13]). Regarding to how were the rates of distance covered during effective playing time calculated: i) the distances covered during effective playing time were considered; ii) the rates of distance covered during effective playing time of every match normalized to meters per unit of time (m/min.) were calculated; iii) finally, the mean of this metric for each physical variable (TD, MSR, HSR, etc…) was calculated. The total playing time was defined as the duration of the match as a whole, including injury time. The effective playing time refers to the duration of play after subtracting the time taken up by stoppages, substitutions, injuries, and goals [15].

### Statistical Analysis

All statistical analyses were conducted using R–Studio [23]. Considering the characteristics of the sample, organized hierarchically, nested in groups, and with a longitudinal structure, we considered that the best procedure to analyze the data was through Linear Mixed Models (LMM). LMM were used to analyze the effects of competitive standards on player activity normalized by effective playing time or total time. First, a two-level hierarchy was modeled for the analysis. The match physical demands variables (i.e., distances covered at different speed thresholds) were included as dependent variables in the models, and leagues (First and Second Division) and tiers (Tiers A, B, C, D, and E) were the independent variables included as fixed effects. The team variable was considered as the random effect in the analysis. Values were represented as coefficients and standard error (Coeff ± SE). Statistical significance was set at *p* < .05. Finally, Cohen’s d effect size was calculated and interpreted as follows: 0.00–0.19: trivial; 0.20–0.59: small; 0.60–1.19: moderate; 1.20–1.99: large; ≥ 2.00: very large [24].

## RESULTS

### Effective Playing Time

The mean effective playing time was significantly higher in matches of the First Division than in the Second Division (52 minutes 54 seconds ± 21 seconds vs. 51 minutes 54 seconds ± 20 seconds, *p* < .01). The mean effective playing time in matches played by Tier A (55 minutes 36 seconds ± 50 seconds) was significantly higher compared to Tier C (52 minutes 18 seconds ± 1 minutes, 10 seconds; *p* < .01), D (53 minutes 6 seconds ± 43 seconds; *p* < .05), and E (51 minutes 30 seconds ± 21 seconds; *p* < .01).

### Match Running Performance

**FIG. 1:**
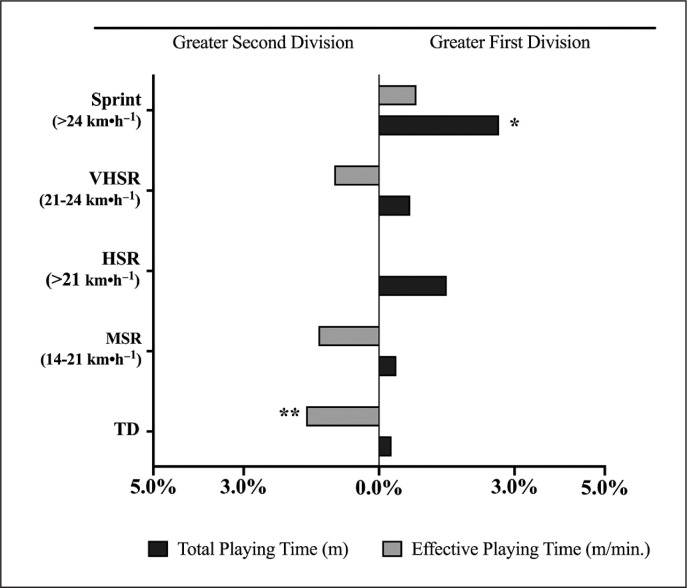
Percentage of change in match running performance between First and Second Division by total or effective playing time. Note: TD = total distance; MSR = medium-speed running; HSR = high-speed running; VHSR = very high-speed running; Sprint = sprinting speed running distance. *p < .05; **p < .01.

Descriptive statistics of both competitive standards are summarized in Table 1, whereas Figure 1 depicts the differences in percentage from the First Division to the Second Division. Taking into account the total playing time, First Division teams covered significantly higher sprinting distance (*p* < .05) than teams from the Second Division. No significant differences were found between both competitive standards on TD, MSR, HSR and VHSR distances.

**TABLE 1:** Match running performance differences between leagues considering effective and total playing time.

	LaLiga Santander		LaLiga Smartbank		*p*	*d* (CI_95%_)

	*Coeff*	*SE*	*Coeff*	*SE*		
TD (m)	110,544	356	110,238	331	.387	.01
TD/min. effective (m × min^−1^)	2,104	13.8	2,138	12.9	.009	.36
MSR (m)	23,476	211	23,386	199	.623	.04
MSR/min. effective (m × min^−1^)	447	4.73	453	4.48	.127	.22
HSR (m)	6,338	72.0	6,243	67.3	.171	.37
HSR/min. effective (m × min^−1^)	121	1.54	121	1.43	.848	.14
VHSR (m)	3,182	34.2	3,160	31.9	.510	.24
VHSR/min. effective (m × min^−1^)	60.6	0.71	61.2	0.66	.346	.03
Sprint (m)	3,163	42.2	3,079	39.2	.046	.41
Sprint/min. effective (m × min^−1^)	60.3	0.90	59.8	0.83	.586	.21

*Note*. Coeff = Coefficient; SE = Standard Error; m = meters; m × min^−1^ = meters per minute; TD = Total distance covered; MSR = Medium-speed running; HSR = High speed running; VHSR = Very high-speed running; Sprint = Sprint speed running distance.

However, when the effective playing time was considered, there were no differences in sprinting distance between First and Second Division. Contrarily, teams from the First Division covered significantly less TD compared with teams from the Second Division (*p* < .05). No significant differences were found between both competitive standards on MSR, HSR and VHSR distances.

### Contextualised match running performance according to tiers

Table 2 shows the differences between ranking groups on match running performance. Taking into account the total playing time, Tiers E teams covered significantly less HSR compared to Tier A (*p* < .05) and C (*p* < .01); less VHSR than Tier C (*p* < .05); and less Sprint than Tier A (*p* < .05), C (*p* < .01), and D (*p* < .05). However, when the effective playing time was considered, Tier E teams covered significantly more TD compared to Tier A (*p* < .01) and D (*p* < .05); and Tier C teams covered significantly more sprinting distance compared to Tier E (*p* < .05). No significant differences were found between both competitive standards on MSR, HSR, and VHSR distances.

**TABLE 2:** Match running performance differences between ranking groups considering effective and total playing time.

Variables	Tier A			Tier B			Tier C			Tier D			Tier E		

	*Coeff*	*SE*	*p*	*Coeff*	*SE*	*p*	*Coeff*	*SE*	*p*	*Coeff*	*SE*	*p*	*Coeff*	*SE*	*p*
Effective minutes	55.6	.89	c^**^, d^*^, e^***^	53.1	1.02		52.3	.58	a^**^	53.1	.72	a^*^	51.5	.35	a^***^

TD (m)	111,314	1,007		109,399	1,163		110,617	649		110,177	789		110,294	397	

TD/min. effective (m × min^−1^)	2,015	36.8	c^*^, e^**^	2,071	42.5		2,127	23.7	a^*^	2,086	28.8	e^*^	2,153	14.5	a^**^, d^*^

MSR (m)	23,876	630		22,886	728		23,771	394		23,529	464		23,289	248	

MSR/min. effective (m × min^−1^)	433	14.05		434	16.22		457	8.78		444	10.35		454	5.53	

HSR (m)	6,590	197.3	e^*^	6,421	227.8		6,518	126.8 e^**^	6,440	153.6		6,118	77.7	a^*^, c^**^

HSR/min. effective (m × min^−1^)	119	4.32		122	4.99		125	2.78		122	3.38		119	1.70	

VHSR (m)	3,300	94.1		3,157	108.7		3,273	60.7	e^*^	3,203	73.7		3,111	37.1	c^*^

VHSR/min. effective (m × min^−1^)	59.8	2.00		59.9	2.31		62.9	1.29		60.7	1.57		60.7	0.79	

Sprint (m)	3,290	114.1	e^*^	3,264	131.8		3,244	74.0	e^**^	3,237	90.4	e^*^	3,008	45.0	a^*^, c^**^, d^*^

Sprint/min. effective (m × min^−1^)	59.6	2.50		62.0	2.88		62.5	1.62	e^*^	61.6	1.98		58.8	0.99	c^*^

*Note*. Coeff = coefficient; SE = Standard Error; m = meters; m × min^−1^ = meters per minute; TD = Total distance covered; MSR = Medium-speed running; HSR = High speed running; VHSR = Very high-speed running; Sprint = Sprint speed running distance; a = significant differences compared with Tier A; b = significant differences compared with Tier B; c = significant differences compared with Tier C; d = significant differences compared with Tier D; e = significant differences compared with Tier E;

* *p* < .05;

** *p* < .01;

*** *p* < .001.

## DISCUSSION

The aim of the present study was to compare the match running performance of players in the top two competitive standards of Spanish soccer considering the effective playing time of matches. The main findings of the study reported that: (a) the effective playing time was significantly higher in matches of the First Division than in the Second Division, (b) when the total playing time was considered, First Division teams covered significantly higher sprinting distance than teams from the Second Division, (c) however, when the effective playing time was considered, there were no differences in sprinting distance between First and Second Division. Contrarily, teams from the First Division covered significantly less TD compared with teams from the Second Division. No significant differences were found between both competitive standards on MSR, HSR and VHSR distances.

Contrary to previous research which showed differences in different competitive standards [3, 6–8], this study did not find relevant differences on match running performance when the effective playing time was considered. Only the total distance covered was different for the first and second division teams. As hypothesized, the differences in players’ match running performance in different competitive standards may be influenced by differences in the effective playing time of games. Previous research demonstrated that the effective playing time is higher in highest competitive standards and consequently matches in the highest standards are more fluid than in lower levels [14]. For example, the difference in the percentage of effective playing time between the top two competitive standards in Italy, Spain, England, France, and Germany was 3.7%, 2.8%, 3.3%, 1.5% and 4.3%, respectively [14]. Our results strongly support this finding. According to the current results, the effective playing time is 2% higher in matches of the First Division. Consequently, it seems that variations on the workload of the players could not be linked to the competitive standards of the players. Rather, it is related to differences in the effective playing time of matches.

Match analysis has shown that sprint and high-intensity efforts are the most important physical actions in soccer due to the relationship with training status and their ability to discriminate between different levels of play [25, 26]. Our results showed, under the total playing time condition, that First Division teams covered significantly higher sprinting distance than teams from the Second Division, which is likely due to significantly greater effective playing time during matches of the First Division, and the majority of sprint distance (97%) being covered during effective playing time [18]. These results are similar to those provided in previous studies. However, considering the effective playing time and normalizing the distance covered to meter per unit of time (m · min^−1^), the current results found that there are no significant differences between both competitive standards on MSR, HSR, VHSR, and sprint distances; and teams from the First Division covered significantly less TD. This suggests that the effective playing time gives a more representative overview of soccer players’ match running performance, indicating that the total playing time could erroneously estimate match running performances in soccer.

Considering the strength of the teams according to their final league ranking, the effective playing time was lower as Tiers were lower. For example, the difference in the effective playing time between Tier A (55.6 ± .89 minutes) and Tiers B (53.1 ± 1.02 minutes), C (52.3 ± .58 minutes), D (53.1 ± .72 minutes) and E (51.5 ± .35 minutes) was + 2.5, + 3.3, + 2.5, and + 4.1 minutes, respectively. Again, it seems that matches in the highest standards are more fluid than in lower levels [14]. In fact, playing against highest quality teams involves significantly greater TD covered by teams [27]. Overall, no differences on match running performance were found between tiers according to their final league ranking. These results are different to those provided by previous research [19, 28–30]. Probably this may be due to the fact that in these studies the effective playing time was not considered. Additionally, future studies should contextualize match running performance considering simultaneously tactical and technical data. As it has been suggested recently [28], scientific literature have to understand why and how teams and players cover more or less distance depending on the game context.

Concerning the limitations of the current study, some aspects should be acknowledged: (a) previous evidence showed that physical performance of soccer players may be affected by their age. Thus, this variable should be considered in further studies; (b) different contextual-related variables, like opponent quality or scoreline [27, 31] or the playing style of each tier teams [32] should be included in future studies; (c) match performance variables should be individualized for each player rather than a general speed threshold for all of them; (d) finally, it should be noted that matches can be clustered based on the residual time and then increase the utility of the normalized data.

These findings may have a great deal of practical implications and may help coaches and strength and conditioning specialist to better understand match running performance in elite soccer. To achieve a comprehensive analysis of the real physical demands of official soccer matches, practitioners should take into account the effective playing time, as variations on the workload of the players could not only be linked to the competitive standards of the players. That means, the practitioners could know the exact workload of the players on matches considering the effective playing time and normalizing the distance covered to meter per unit of time (m · min^−1^). This strategy can provide information to prescribe effective stimuli to optimize the physical performance of soccer players in the training sessions [18].

## CONCLUSIONS

In conclusion, to our knowledge this is the first study to date that compares the match running performance of players across different competitive standards accounting for effective playing time. The data demonstrate that the distance covered by teams was similar in lower and higher competitive standards when game interruptions are considered. Finally, future studies examining the differences in physical metrics between competitive standards should calculate the physical metrics as a rate considering effective playing time.

## Supplementary Material

Match running performance is similar in lower and higher competitive standards of Spanish professional soccer accounting for effective playing time

## Data Availability

Restrictions apply to the availability of these data. Data was obtained from LaLiga and are available at https://www.laliga.es/en with the permission of LaLiga.
