# Supplementary material for: Match running performance is similar in lower and higher competitive standards of Spanish professional soccer accounting for effective playing time
Source: Biol Sport. 2023 Dec 20;41(3):39–46. doi: 10.5114/biolsport.2024.132993 (PMC11167472; doi:10.5114/biolsport.2024.132993)
Supplement: Match running performance is similar in lower and higher competitive standards of Spanish professional soccer accounting for effective playing time [file JBS-41-51822-s1.pdf]

SUPPLEMENTAL TABLE 1. Match physical demands differences between leagues considering effective and total playing time.

	LaLiga Santander		LaLiga Smartbank		*p*
	Mean	***SD***	***Mean***	***SD***	
TD (m)	110,544	356	110,238	331	.387
TD effective (m)	83,030	488	81,465	452	.001
MSR (m)	23,476	211	23,386	199	.623
MSR effective (m)	21,989	202	21,699	191	.108
HSR (m)	6,338	72.0	6,243	67.3	.171
HSR effective (m)	6,224	71.5	6,110	66.8	.096
VHSR (m)	3,182	34.2	3,160	31.9	.510
VHSR effective (m)	3,102	33.9	3,065	31.6	.263
Sprint (m)	3,163	42.2	3,079	39.2	.046
Sprint effective (m)	3,129	41.9	3,040	38.9	.034

Note. SD = Standard Deviation; m = meters; TD = Total distance covered; MSR = Medium-speed running; HSR = High speed running; VHSR = Very high-speed running; Sprint = Sprint speed running distance.

SUPPLEMENTAL TABLE 2. Match physical demands differences between quality ranking groups considering effective and total playing time.

Variables	Tier A			Tier B			Tier C			Tier D			Tier E		
	Mean	***SD***	***p***	***Mean***	***SD***	***p***	***Mean***	***SD***	***p***	***Mean***	***SD***	***p***	***Mean***	***SD***	***p***
TD (m)	111,314	1,007		109,399	1,163		110,617	649		110,177	789		110,294	397	
TD effective (m)	85,647	1,316	e**	82,395	1,519		82,605	857		82,922	1,050		81,272	519	a**
MSR (m)	23,876	630		22,886	728		23,771	394		23,529	464		23,289	248	
MSR effective (m)	22,404	600		21,440	693		22,215	377		22,021	447		21,618	236	
HSR (m)	6,590	197.3	e*	6,421	227.8		6,518	126.8	e**	6,440	153.6		6,118	77.7	a*, c**
HSR effective (m)	6,467	196.1	e*	6,323	226.4		6,399	126.0	e**	6,319	152.6	e*	5,986	77.3	a*, c**, d*
VHSR (m)	3,300	94.1		3,157	108.7		3,273	60.7	e*	3,203	73.7		3,111	37.1	c*
VHSR effective (m)	3,216	93.6	e*	3,086	108.1		3,190	60.3	e*	3,117	73.3		3,018	36.9	a*, b*
Sprint (m)	3,290	114.1	e*	3,264	131.8		3,244	74.0	e**	3,237	90.4	e*	3,008	45.0	a*, c**, d*
Sprint effective (m)	3,251	113.4	e*	3,236	130.9		3,208	73.5	e**	3,202	89.7	e*	2,969	44.7	a*, c*, d*

Note. SD = Standard Deviation; m = meters; TD = Total distance covered; MSR = Medium-speed running; HSR = High speed running; VHSR = Very high-speed running; Sprint = Sprint speed running distance; a = significant differences compared with Tier A; b = significant differences compared with Tier B; c = significant differences compared with Tier C; d = significant differences compared with Tier D; e = significant differences compared with Tier E; $^{*}p < .05$; $^{**}p < .01$; $^{***}p < .001$.
